# Maternal and Neonatal Outcomes among Pregnant Women with 2009 Pandemic Influenza A(H1N1) Illness in Florida, 2009-2010: A Population-Based Cohort Study

**DOI:** 10.1371/journal.pone.0079040

**Published:** 2013-10-24

**Authors:** Timothy J. Doyle, Kate Goodin, Janet J. Hamilton

**Affiliations:** 1 Florida Department of Health, Bureau of Epidemiology, Tallahassee, Florida, United States of America; 2 Centers for Disease Control and Prevention, Atlanta, Georgia, United States of America; National Institute for Public Health and the Environment, Netherlands

## Abstract

**Introduction:**

Pregnant women have been identified as a high risk group for severe illness with 2009 pandemic influenza A(H1N1) virus infection (pH1N1). Obesity has also been identified as a risk factor for severe illness, though this has not been thoroughly assessed among pregnant women. The objectives of this study were to provide risk estimates for adverse maternal and neonatal outcomes associated with pH1N1 illness during pregnancy and to assess the role of obesity in these outcomes.

**Methods:**

We established a retrospective population-based cohort of all live births occurring in Florida during the first 15 months of the pandemic. Illness with pH1N1 during pregnancy was ascertained through record linkage with the Florida state notifiable disease surveillance database. Data from the birth record, including pre-pregnancy body mass index, were analyzed to assess risk of adverse outcomes associated with pH1N1 illness.

**Results:**

A total of 194 women were identified through surveillance with pH1N1 illness during pregnancy. Children born to women with pH1N1 illness during pregnancy were at increased risk for low birth weight [OR (95%CI): 1.78 (1.11-2.860)], premature birth [2.21 (1.47-3.330)], and infant death [4.46 (1.80-11.00)], after adjusting for other factors. Women with pH1N1 illness during pregnancy were at increased risk for severe outcomes including admission to an intensive care unit. Obesity was an observed risk factor, both for the more severe pH1N1 illness detected through surveillance, and for severe maternal outcomes.

**Conclusions:**

Case-patients in this analysis likely represent the most severely ill subset of all women infected with pH1N1 during pregnancy, limiting the generalizability of these findings to more severely ill patients rather than influenza infection in general. Nevertheless, these results suggest that more severe pH1N1 illness during pregnancy is associated with adverse neonatal outcomes and that pregnant women should continue to be targeted for appropriate prophylaxis and early treatment.

## Introduction

Infection with influenza virus can result in a spectrum of illness ranging from asymptomatic infection to severe illness and death. Soon after recognition of the novel 2009 pandemic influenza A(H1N1) virus (pH1N1), pregnant women were identified as a high risk group for severe illness [1,2]. Severe illness may be due to respiratory, immunologic, or other physiologic changes that normally occur during pregnancy [3]. Numerous additional reports have further described increased risk for adverse maternal and neonatal outcomes among pregnant women infected with pH1N1[4–12]. Many of these reports called for additional prospective studies with larger sample sizes, robust comparison groups, longer follow-up periods, and additional control for potential confounding factors.

Florida is the fourth most populous state in the U.S. with an estimated population in 2010 of more than 18.8 million residents [13]. More than 200,000 live births occur in the state each year [14]. For each live birth, the birth record contains information regarding baseline demographic and health information of the birth mother, including pre-pregnancy body mass index (BMI), exposures during pregnancy such as tobacco and alcohol use, and maternal and neonatal morbidity indicators such as admission to an intensive care unit (ICU).

The 2009 influenza pandemic began early in Florida, with cases identified in April 2009 and more than 1430 cases reported by June 30, 2009 [unpublished surveillance data]. The summer of 2009 was characterized by persistent transmission across the state, amplified by localized outbreaks in group settings. Following resumption of school, peak transmission was observed in October 2009 [15]. In a national case series of pregnant women with confirmed pH1N1 illness in 2009, Florida had the second highest number of cases, following California [11]. 

In several of the initial case series of pregnant women with pH1N1 illness, data on birth outcomes was incomplete, and study designs and available data often did not permit control for other factors associated with adverse neonatal outcomes. Longer term follow-up was needed to ascertain pregnancy outcomes, as well as to fully characterize the impact on infant mortality beyond the perinatal period. Population cohort designs, derived from birth certificate data, may permit more precise risk estimates through a population-based comparison group and better control for confounding. In particular, obesity has been previously identified not only as a risk factor for severe pH1N1 illness[16–18], but also as a risk factor for maternal [19] and infant death [20,21]. Pregnant women were excluded from some past studies identifying obesity as a risk factor for severe pH1N1 illness, however, as data were not always available for pre-pregnancy BMI. The objectives of this study were to generate more precise risk estimates for adverse maternal and neonatal outcomes associated with pH1N1 illness identified during pregnancy, and to further explore the role of obesity in these outcomes. To do this, we analyzed data from a retrospective population-based cohort of live births in Florida occurring during the period 2009-2010. 

## Methods

Prior to the recognition of 2009 pH1N1, infection with a novel influenza virus was already a reportable condition in Florida [22]. Thus, as soon as the pandemic began, health providers in Florida were already required to report all individual cases to the Florida Department of Health (FLDOH). Initially, all laboratory confirmation of pH1N1 in the United States was conducted by the Centers for Disease Control and Prevention (CDC). In May 2009, Florida state public health laboratories became certified for confirmatory testing and later that summer, commercial labs also became certified. Case confirmation required positive test results from viral culture or real-time reverse-transcriptase polymerase chain reaction (rRT-PCR) assays, using primers specific to the novel virus. 

On August 3, 2009, the state issued new guidance to health care providers and laboratories to test for influenza and, if positive for pH1N1, report people with influenza that was life-threatening or resulted in death, or resulted in hospitalization of a pregnant woman. Providers and facilities were also asked to report people who were part of clusters or outbreaks of influenza-like illness. Despite the change in disease reporting guidelines, some providers continued for several weeks to report non-hospitalized cases of pandemic influenza that were laboratory confirmed. All individual case reports received by the FLDOH meeting the case definition of an acute respiratory illness, with laboratory confirmation of pH1N1 by a certified laboratory, were maintained in the state’s notifiable disease surveillance database. Illness on the more severe end of the spectrum is more likely to be apparent, lead to specific laboratory testing, and be captured through passive notifiable disease surveillance [23]. Standard data contained in the case reports included patient identifying and demographic information, pregnancy status of individuals at the time of their illness, information regarding treatment with antiviral medication, presence of co-morbid conditions, and outcome of illness.

The Florida birth registry is maintained by the FLDOH Office of Vital Statistics and includes records on all live births occurring in Florida. Records are submitted by hospitals and other medical providers and contain information on the newborn, as well as the mother and father. There are >330 variables contained in the comprehensive electronic birth record, including information on maternal and infant demographic variables; pre-pregnancy BMI, pre-natal exposures such as alcohol and tobacco use by the mother; obstetric history, medical care received during labor and delivery; and health status of the infant. For this analysis, we also used a variable indicating if the mother resided within the city limits, or in a more rural location outside the city limits. In addition, the electronic birth record is updated to indicate mortality for any child dying within five years following live birth. 

Electronic records of live births from the Florida birth registry were cross-referenced to the state notifiable disease database to identify children born to women with pH1N1 illness during their pregnancy. All pH1N1 confirmed case-patients with onset of illness between April 24, 2009 and May 31, 2010, who were pregnant at the time of their illness, were included. The record matching included a combination of automated methods and manual review, in an iterative process. Firstly, records in the birth registry were matched to the disease surveillance database by the mother’s social security number and date of birth. Other combinations of matching variables subsequently used included mothers first and last name, alone or in combination with social security number, or date of birth. All matched records were manually reviewed to ensure the accuracy of the match.

Data were further compiled from de-identified records of a birth cohort of all live births occurring in Florida between June 1, 2009 and September 1, 2010. This period corresponds with the time interval of live births among women with reported pH1N1 illness. Records for women with multiple gestational (plural) pregnancies were identified and later de-duplicated to also permit analysis of the cohort of pregnant women giving birth during the same interval. Records were excluded for births among non-Florida residents, and in instances in which adoption or foundling was indicated on the birth record. The database was updated in January 2012 to reflect any infant deaths following birth, occurring up to that time. For secondary analysis, an alternative pregnancy and birth comparison cohort was also constructed from the pre-pandemic period to include all live births occurring between January 1 and February 28, 2009. 

Illness due to pH1N1 infection during pregnancy was treated as the outcome variable when assessing disease risk and treated as the primary exposure variable for assessing risk of adverse maternal and neonatal outcomes. Women and children in the pregnancy and birth cohorts were categorized as pH1N1 illness in pregnancy (i.e. ‘ill’ for the first analysis or ‘exposed’ for the second) if the birth records matched to the pH1N1 notifiable disease surveillance database by the methods previously described. All women and their newborns who did not match to the disease surveillance database were defined as ‘unexposed’ or non-cases of pH1N1 illness during pregnancy. Measures of association between exposure and outcome were computed using univariate and multivariable logistic regression and expressed as odds ratios (OR) with 95 percent Wald confidence intervals (95% CI). Univariate relative risks (RR) were also computed for comparison with ORs, but were not presented in the results tables. Because minimal differences were generally noted between the univariate RRs and ORs and because outcome frequencies were generally uncommon and less than 10%, odds ratios were used throughout as approximate estimates of relative risk. To adjust for possible confounding, standard and stepwise multivariable logistic regression was used. Multivariable models were constructed using variables identified during univariate analyses as being associated with influenza, or known risk factors for adverse maternal and neonatal outcomes, while also attempting to minimize the exclusion of cohort members due to missing data for a particular variable. For all stepwise analysis, α=0.1 was the threshold used for variable entry into the model, and α=0.05 was used for retaining variables in the final model. Data analysis was conducted using SAS software, version 9.1 [SAS Institute; Cary, NC]. Statistical power computations presented in the results were obtained using the computer program Power v3.0 [National Institute of Health, National Cancer Institute].

Data on pre-pregnancy BMI was available for approximately 94% of records and was expressed as [weight in kilograms/(height in meters)^2^]. BMI data in the Florida birth registry has previously been found to be generally reliable and valid [24]. Nevertheless, the top and bottom 0.5% of BMI values were excluded as non-valid, resulting in the inclusion of BMI values only between 16.2 and 49.1. Standard cut points were used to categorize women based on pre-pregnancy BMI [25] and analyses were conducted using both 4 and 5 different categories. For analysis using 4 categories, the BMI cut points were as follows: underweight (<18.5), normal (18.5-24.9), overweight (25.0-29.9), and obese (≥ 30.0). For analysis using 5 categories of BMI, the obese grouping was further divided into obese (30.0-34.9) and very obese (≥ 35.0). Analyses were also conducted using BMI as a continuous variable. Since pre-pregnancy BMI was not available for approximately 6% of women in the cohort, analyses were conducted both with and without BMI in the models. Interaction between pH1N1 illness and obesity was also assessed in relation to the most severe maternal and neonatal outcomes by including in the logistic model, the first level interaction term between pH1N1 illness and obesity (yes/no), defined as pre-pregnancy BMI ≥ 30.0. 

For simplicity, adult BMI categories were used throughout the analysis, despite the fact that some gravida in the analysis were <20 years old, and standard classifications for BMI are different for children and adolescents [26]. Maternal age at time of birth was alternatively treated as either a continuous variable, dichotomized as ≤ 25 and >25 years, or classified into 3 groups consistent with previous analyses using Florida birth registry data (<18, 18-39, ≥ 40) [21]. The last grouping is generally consistent with accepted risk categories for adverse maternal and fetal outcomes. Continuous variables such as birth weight and gestational age at birth were categorized using generally accepted cut points to assess adverse neonatal outcomes. Admission to an Intensive Care Unit (ICU) was used as an indicator of severity of illness for both adverse maternal and neonatal outcomes. 

Data regarding influenza vaccination history and antiviral treatment for influenza was missing from the surveillance case reports for a large proportion of case-patients. Furthermore, the majority of case-patients had illness onset before a vaccine specific for pH1N1 became available. Therefore, we did not attempt in this analysis to assess the role of vaccination or antiviral treatment for influenza on maternal and neonatal outcomes.

### Ethics Statement

This activity was reviewed by the Ethics and Human Research Protection Program of the FLDOH, and determined to involve analysis of existing and de-identified data, not human subjects research, and was therefore exempt from further review by an institutional review board.

## Results

**Figure 1:**
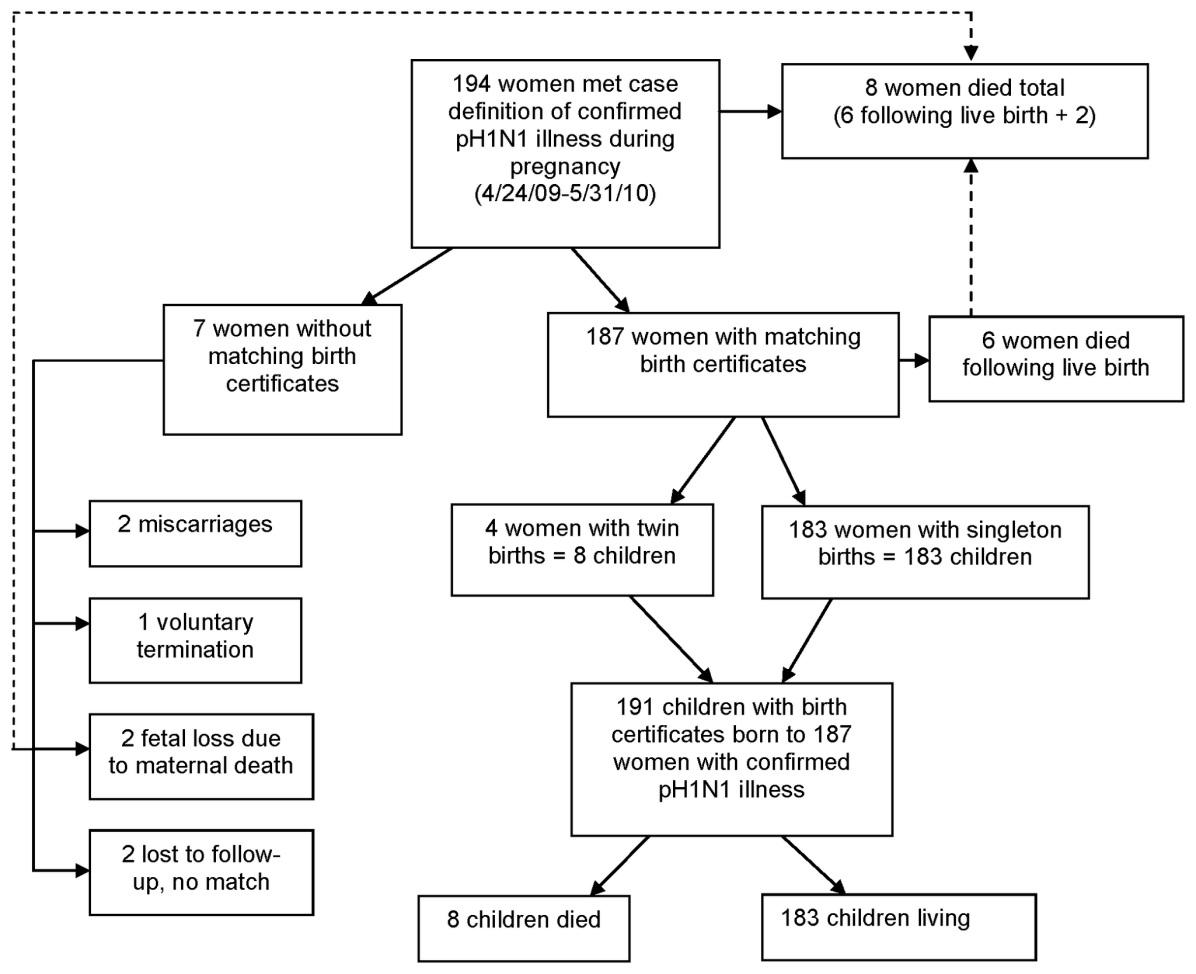
Distribution of case-patients by vital status and birth record matching.

A total of 194 women with confirmed pH1N1 illness during pregnancy were reported to FLDOH. Of these, matching birth certificates were identified for 187 women (96%); 4 women gave birth to twins, resulting in a total of 191 live births (Figure 1). Of these 191 children, 8 died following live birth. Of the 194 pregnant women with pH1N1 illness, 8 (4%) women died; 6 following live birth of their infants. Seven of the 194 pregnant pH1N1 case-patients did not have corresponding birth certificates. Two of these women were lost to follow-up without a matching birth certificate, and a live birth did not occur for the other 5 women: 2 pregnancies ended in miscarriage, 2 ended in fetal loss secondary to maternal death, and 1 pregnancy was voluntarily terminated (Figure 1).

**Figure 2:**
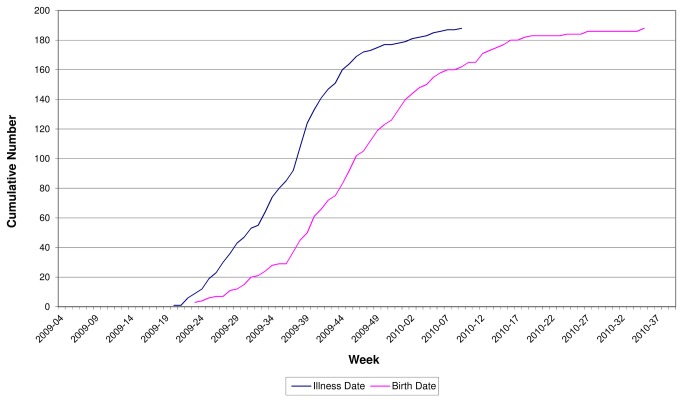
Cumulative pandemic influenza illness in pregnant women by illness and delivery date.

Among pH1N1 case-patients, the temporal pattern of cumulative onset of influenza illness and subsequent birth is shown in Figure 2. Of the 194 pregnant case-patients, approximately 24% had symptom onset and laboratory confirmation prior to August 2009, when disease reporting guidelines were changed. The number of cases declined rapidly after November 2009. Of the 194 women with pH1N1 illness identified through surveillance, 24 (12%) were not hospitalized for their influenza illness. Onset of illness for non-hospitalized case-patients ranged from June 2009 to February 2010, with more than two-thirds having illness onset after August 2009.

Non-pH1N1 associated individuals in the birth and pregnancy cohort included, after exclusions, 300,398 children born to 295,747 women between June 1, 2009 and September 1, 2010. A total of 4,198 birth records were excluded from the cohort due to non-Florida residence of the mother (n=1,547), child adopted (n=2,648), or foundling (n=3). The alternative comparison birth and pregnancy cohort from the pre-pandemic period included, after exclusions, 24,008 children born to 23,679 women.

### Analysis of risk factors associated with pH1N1 illness reported through surveillance

Among the cohort of pregnant women giving birth between June 1, 2009 and September 1, 2010, univariate analysis indicated increased risk of pH1N1 illness detected through surveillance among non-white women, and those of Hispanic or Haitian ethnicity. Increased risk of detected and reported pH1N1 illness was also associated with being 25 or younger, unmarried, high school graduate or less education, being on Medicaid, receiving Women Infants and Children (WIC) Program food supplements, living within the city limits, having one or more previous live births, and being obese (Table 1). Influenza illness risk was associated with pre-pregnancy BMI category in a dose-response fashion (Mantel-Haenszel test for trend, 4 categories of BMI, p=0.02; 5 categories of BMI, p=0.01). In the multivariable stepwise regression model, the factors most strongly associated with pH1N1 illness were younger age, residence within city limits, Hispanic or Haitian ethnicity, previous live births, lower education status, and increasing pre-pregnancy BMI (Table 2).

**Table 1:** Demographic and other characteristics of cases and non-cases of pandemic influenza illness, as ascertained from child’s birth certificate.

**Characteristics of women**	**Women with confirmed pH1N1 illness during pregnancy, n (%)**	**Women without confirmed pH1N1 illness during pregnancy, n (%)**	**Unadjusted OR (95% CI)**
**Total pregnant women**	187	295,747	
**Race**			
Non-white	95 (51)	112,968 (38)	1.67 (1.25-2.23)
white	92 (49)	182,779 (62)	1.0 (ref)
**Ethnicity**			
Hispanic or Haitian	86 (47)	94,253 (32)	1.88 (1.41-2.52)
Not Hispanic or Haitian	96 (53)	198,249 (68)	1.0 (ref)
**Foreign born**			
yes	69 (37)	92,053 (31)	1.29 (0.96-1.74)
no	118 (63)	203,694 (69)	1.0 (ref)
**Age at delivery of child**			
≤ 25	104 (56)	117,923 (40)	1.89 (1.42-2.52)
>25	83 (44)	177,800 (60)	1.0 (ref)
Mean	25.9	27.6	p<0.01*
**Marital status**			
single	115 (62)	139,315 (47)	1.79 (1.34-2.41)
married	72 (38)	156,337 (53)	1.0 (ref)
**Education**			
High school graduate or less	123 (68)	144,650 (49)	2.16 (1.58-2.94)
High school education	59 (32)	149,734 (51)	1.0 (ref)
**Pre-pregnancy BMI**			
<18.5	5 (3)	12,065 (4)	0.77 (0.31-1.90)
18.5-24.9	74 (44)	136,848 (50)	1.0 (ref)
25.0-29.9	41 (25)	69,260 (25)	1.10 (0.75-1.60)
≥ 30.0	46 (28)	56,194 (21)	1.51 (1.05-2.19)
Mean	26.9	25.7	p<0.01*
**Principle source of payment**			
Medicaid	112 (60)	140,680 (48)	1.63 (1.22-2.18)
Private insurance, self, or other	75 (40)	153,592 (52)	1.0 (ref)
**Received WIC food**			
Yes	126 (68)	154,191 (53)	1.95 (1.43-2.66)
No	58 (32)	138,090 (47)	1.0 (ref)
**Residence in city limits**			
Yes	155 (83)	210,535 (72)	1.95 (1.33-2.87)
No	31 (17)	82,161 (28)	1.0 (ref)
**Previous live births**			
Yes	127 (68)	168,940 (57)	1.59 (1.17-2.16)
No	60 (32)	126,807 (43)	1.0 (ref)
**>2 Other children (live births**)			
Yes	28 (15)	29,028 (10)	1.62 (1.08-2.42)
No	159 (85)	266,719 (90)	1.0 (ref)
**Multiple gestation**			
Multiple	4 (2)	5064 (2)	1.26 (0.47-3.38)
Single	183 (98)	290,666 (98)	1.0 (ref)

* t-test, pooled variance

**Table 2:** Multivariable analyses of demographic and other characteristics associated with pandemic influenza illness during pregnancy.

**Characteristics of Pregnant Women**	**Adjusted OR^1^**	**95% CI**	**Adjusted OR^2^**	**95% CI**
Age (years)	0.95	(0.93-0.98)	0.95	(0.92-0.98)
Residence in city limits	2.14	(1.42-3.23)	1.94	(1.28-2.93)
Hispanic or Haitian ethnicity	1.77	(1.32-2.39)	1.88	(1.37-2.57)
Other children (live births)	1.75	(1.26-2.45)	1.67	(1.18-2.38)
High school graduate or less	1.55	(1.10-2.17)	1.47	(1.03-2.10)
Pre-pregnancy BMI	n/a	n/a	1.03	(1.01-1.06)

^1^ Final model included 178 cases and 287,449 non-cases with available data for all variables (8307 observations deleted for missing data on one or more variables). Multivariable stepwise logistic regression model adjusting for maternal age, maternal education, mother residence in city, maternal ethnicity, presence of past live births, maternal marital status, maternal race, maternal foreign born, and Medicaid payment. All variables not listed in the table were not significant at alpha=0.05 level, after adjusting for other variables, and thus did not appear in the final model.

^2^ Final model included 160 cases and 267,880 non-cases with available data for all variables (27,894 observations deleted for missing data on one or more variables). Multivariable stepwise logistic regression model adjusting for same variables in model 1, with addition of maternal pre-pregnancy BMI (continuous variable). All variables not listed in the table were not significant at alpha=0.05 level, after adjusting for other variables, and thus did not appear in the final model.

When comparing the distribution of women with pH1N1 illness by variables listed in Table 1, to the distribution of women in the alternate comparison group who gave birth in the pre-pandemic period, the results were remarkably similar. Pregnant women with pH1N1 illness were more likely than pregnant women in the pre-pandemic period to be non-white, Hispanic or Haitian, non-married, obese, age 25 or under, with lower educational attainment, receiving public assistance, living within city limits, and with previous live births (data not shown).

### Analysis of neonatal outcomes

Neonatal outcomes among the cohort of live births occurring between June 2009 and September 2010, are shown in Table 3. Children born to women with pH1N1 illness during pregnancy were at increased risk for preterm birth, low birth weight, 5-minute APGAR score <9, admission to a neonatal ICU, and death. Children born to women with pH1N1 illness were at 4 to 5 times increased risk of infant death than those born to non-ill mothers. The mean birth weight of infants born to pH1N1 ill women was 200 grams less than those born to non-ill women (3,030g versus 3,229g; t-test, p<0.001) and the mean gestational age at birth was one week less (37.3 weeks versus 38.3 weeks; p<0.001). The mean 5-minute APGAR score of pH1N1 associated infants was 8.56, versus 8.84 for all other infants born during the interval (p<0.001). The data also suggest that women with pH1N1 illness, as a group, were somewhat more likely to require emergency cesarean section delivery. Similar patterns were observed when comparing birth outcomes using the alternative pre-pandemic comparison group. We found no evidence of increased risk of congenital anomalies associated with pH1N1 illness during pregnancy. 

**Table 3:** Neonatal outcomes associated with pandemic influenza illness during pregnancy.

**Neonatal outcome**	**pH1N1 during pregnancy,No. (%)**	**No pH1N1 during pregnancy, No. (%)**	**Unadjusted OR (95% CI)**	**Adjusted OR (95% CI)^1^**	**Adjusted OR (95% CI)^2^**
**Low birth weight**					
<2500g	30 (16)	25,998 (9)	1.97 (1.33-2.91)	1.67 (1.06-2.62)	1.78 (1.11-2.86)
2500g or more	161 (84)	274,400 (91)	1.0 (ref)	1.0 (ref)	1.0 (ref)
<1500g	15 (8)	4893 (2)	5.16 (3.05-8.74)	3.66 (1.94-6.91)	3.93 (2.00-7.69)
1500g or more	176 (92)	295,505 (98)	1.0 (ref)	1.0 (ref)	1.0 (ref)
<1000g	6 (3)	2518 (1)	3.84 (1.70-8.66)	2.47 (0.90-6.77)	2.85 (1.03-7.90)
1000g or more	185 (97)	297,880 (99)	1.0 (ref)	1.0 (ref)	1.0 (ref)
**Premature birth**					
<37 weeks gestation	45 (24)	31,259 (10)	2.65 (1.90-3.70)	2.39 (1.64-3.49)	2.21 (1.47-3.33)
37 weeks or more	146 (76)	268,626 (90)	1.0 (ref)	1.0 (ref)	1.0 (ref)
<32 weeks gestation	13 (7)	5152 (2)	4.18 (2.38-7.34)	3.04 (1.57-5.90)	3.21 (1.59-6.48)
32 weeks or more	178 (93)	294,733 (98)	1.0 (ref)	1.0 (ref)	1.0 (ref)
<28 weeks gestation	8 (4)	2258 (1)	5.77 (2.84-11.71)	4.19 (1.82-9.63)	3.95 (1.58-9.90)
28 weeks or more	183 (96)	297,627 (99)	1.0 (ref)	1.0 (ref)	1.0 (ref)
**5-minute APGAR score**					
<9	34 (18)	31,848 (11)	1.82 (1.26-2.64)	1.56 (1.04-2.35)	1.65 (1.08-2.52)
9 or greater	157 (82)	267,851 (89)	1.0 (ref)	1.0 (ref)	1.0 (ref)
**Abnormal condition of newborn**					
yes	36 (19)	37,480 (12)	1.63 (1.14-2.35)	1.34 (0.90-2.00)	1.43 (0.94-2.17)
no	155 (81)	262,918 (88)	1.0 (ref)	1.0 (ref)	1.0 (ref)
**Birth Anomaly**					
yes	2 (1)	3000 (1)	1.05 (0.26-4.23)	1.13 (0.28-4.55)	1.25 (0.31-5.03)
no	189 (99)	297,398 (99)	1.0 (ref)	1.0 (ref)	1.0 (ref)
**Admitted to Neonatal ICU**					
yes	32 (17)	22,975 (8)	2.43 (1.66-3.55)	1.96 (1.27-3.02)	2.14 (1.36-3.36)
no	159 (83)	277,129 (92)	1.0 (ref)	1.0 (ref)	1.0 (ref)
**Mechanical ventilation needed >6 hours**					
yes	3 (2)	1347 (1)	3.54 (1.13-11.09)	1.28 (0.18-9.16)	1.45 (0.20-10.39)
no	188 (98)	298,757 (99)	1.0 (ref)	1.0 (ref)	1.0 (ref)
Neonatal death*					
yes	2 (1)	804 (1)	3.96 (0.98-15.97	4.23 (1.04-17.29	2.43 (0.33-17.65)
no	188 (99)	299,156 (99)	1.0 (ref)	1.0 (ref)	1.0 (ref)
Infant/child death since time of birth**					
yes	8 (4)	2130 (1)	6.12 (3.01-12.44)	5.38 (2.50-11.58)	4.46 (1.80-11.00)
no	183 (96)	297,886 (99)	1.0 (ref)	1.0 (ref)	1.0 (ref)
**Cesarean Delivery**					
yes	92 (48)	115,060 (38)	1.51 (1.14-2.01)	1.44 (1.07-1.94)	1.31 (0.96-1.81)
no	98 (52)	185,148 (62)	1.0 (ref)	1.0 (ref)	1.0 (ref)

^1^ Adjusted for maternal age (<18, 18-39, ≥ 40), maternal race, maternal ethnicity, maternal education, maternal marital status, plurality (y/n), infant sex, tobacco use during pregnancy (y/n), alcohol use during pregnancy (y/n), previous preterm delivery (y/n), previous poor pregnancy outcome (y/n), pre-gestational diabetes (y/n). Depending on outcome variable, between 6582-7318 records were excluded due to missing data for 1 or more variables.

^2^ Adjusted for same variables in model 1, with additional adjustment for pre-pregnancy BMI (4 categories: underweight, normal, overweight, obese). Depending on outcome variable, between 26,675-27,176 records were excluded due to missing data for 1 or more variables.

* Infant non-living at time birth certificate was submitted.

** Infant/child died within 5 years of live birth

Illness with pH1N1 was not significantly associated with low birth weight, after adjusting for gestational age and other variables in the multivariable model. This suggests that the association between pH1N1 illness and low birth weight is primarily due to pre-term birth. No interaction was observed between pH1N1 illness and maternal obesity, with respect to the most severe neonatal outcomes of early preterm birth (<28 weeks, p=0.36), and infant death (p=0.32). 

Of the 191 children born to women with pH1N1 illness, 8 (4%) died (Table 4). This is in addition to the 2 miscarriages and 2 fetal deaths following maternal death (Figure 1). For 2 of the infant deaths following live birth, the mother also died. Most infant deaths following live birth occurred for women infected during the summer of 2009, and during the late second trimester of pregnancy. All but 1 of the 8 children was born premature. Our data capture mortality in children born to pH1N1 infected mothers occurring up to a maximum of 30 months following live birth. Three deaths occurred >1 month following live birth, at 4, 7, and 17 months respectively. Two of these children were born severely premature and their deaths were related to sequelae of premature birth. The other full term infant was born with multiple congenital anomalies including microcephaly, and died of sepsis following surgery and extensive hospitalization. 

**Table 4:** Characteristics of infant and child deaths following live birth to mothers with pandemic influenza illness during pregnancy.

**Onset Symptoms**	**Infant DOB**	**Infant Sex**	**GA at birth (wks)**	**Birth weight (g)**	**Twin**	**Mom age (y)**	**Mom Race**	**Birth Route**	**5 Minute APGARScore**	**Age at death**	**BMI**	**Maternal outcome**
6/21/09	06/23/09	M	27	1150	Yes	25	B	Cesarean	08	17 months	30.2	Alive
6/23/09	07/02/09	M	27	1124	No	22	W	Cesarean	02	<1day	.	D-7/2/09
7/3/09	07/18/09	F	26	936	No	27	W	Cesarean	01	6 minutes	25.8	Alive
8/22/09	10/23/09	M	22	482	No	22	B	Vaginal	08	3 days	19.2	Alive
8/24/09	09/22/09	F	26	595	No	25	W	Cesarean	08	4 month	25.6	Alive
9/6/09	09/17/09	F	24	624	No	31	W-H	Cesarean	06	24 days	25	Alive
11/08/09	11/18/09	M	34	2353	No	22	W-H	Cesarean	03	4 days	.	D-12/8/09
2/4/10	03/29/10	F	38	2637	No	28	W-H	Vaginal	07	7 months	22.3	Alive

M=Male, F=Female, B=Black, W=White, H=Hispanic, D=Deceased, DOB=date of birth, GA=gestational age

### Analysis of maternal outcomes

Based on data available from the birth record, of the 187 women with pH1N1 illness and a live birth, 9 were admitted to ICU. This contrasts with results available from the notifiable disease database, which indicate that 41 (22%) women reported with pH1N1 illness were admitted to ICU. The cause for this discrepancy is unknown, but may be due to the timing of ICU admission following live birth, or incomplete knowledge of the mother’s clinical course by those completing the birth record. Despite this discrepancy, data from the birth record was used to analyze risk of ICU admission for the entire pregnancy cohort. Pregnant women with pH1N1 illness detected through surveillance, were 28 times more likely to be admitted to ICU than other women with a live birth who were not known to be pH1N1 infected (Table 5). The odds of ICU admission associated with pH1N1 illness were reduced slightly to 22.26 after adjusting for other risk factors such as multiple gestational pregnancy, pre-gestational diabetes, previous poor birth outcome, age 40 or over, non-white race, and pre-pregnancy obesity. Extreme obesity, indicated by pre-pregnancy BMI ≥ 35, was associated with a 1.85 times increased risk of ICU admission (95% CI: 1.39-2.48), after adjusting for other factors (data not shown). For adverse maternal outcomes measured by ICU admission, no interaction was observed between pH1N1 illness and obesity (p=0.52).

**Table 5:** Factors associated with maternal admission to Intensive Care Unit (ICU).

**Factor**	**Un-Adjusted OR^1^**	**95% CI**	**Adjusted OR^2^**	**95% CI**	**Adjusted OR^3^**	**95% CI**
pH1N1 illness (Y vs N)	28.28	(14.40-55.55)	22.22	(10.30-47.94)	22.26	(9.69-51.12)
Plurality (Y vs N)			5.27	(3.86-7.20)	5.72	(4.13-7.90)
Pre-gestational diabetes (Y vs N)			3.37	(2.01-5.66)	2.57	(1.40-4.71)
Previous poor outcome (Y vs N)			2.86	(1.64-4.98)	3.17	(1.82-5.53)
Maternal age (≥ 40 vs 18-39)			2.68	(1.94-3.71)	2.51	(1.75-3.59)
Maternal race (non-white vs white)			1.97	(1.66-2.35)	1.83	(1.52-2.21)
Pre-pregnancy BMI (≥ 30 vs normal)					1.52	(1.21-1.92)

^1^ Univariate analysis includes 294,839 pregnant women, 541 of whom were admitted to ICU. Of the 541 admitted to ICU, 9 were pH1N1 infected.

^2^ Multivariable stepwise logistic regression model adjusting for pH1N1 illness, maternal age (<18, 18-39, ≥ 40), maternal race, maternal ethnicity, maternal education, maternal marital status, plurality (y/n), infant sex, tobacco use during pregnancy (y/n), alcohol use during pregnancy (y/n), previous preterm delivery (y/n), previous poor pregnancy outcome (y/n), pre-gestational diabetes (y/n). 6892 observations were deleted for missing data on one or more variables. All variables not listed in the table where not significant at alpha=0.05 level, after adjusting for other variables, and thus did not appear in the final model.

^3^ Adjusted for same variables in model 2, with additional adjustment for pre-pregnancy BMI (4 categories: underweight, normal, overweight, obese). 26,473 observations were deleted for missing data on one or more variables. All variables not listed in the table where not significant at alpha=0.05 level, after adjusting for other variables, and thus did not appear in the final model.

This analysis was repeated after recoding ICU admission to “Yes” for 32 women with pH1N1 illness previously coded “No” in the birth record, but for whom data from the notifiable disease database indicated ICU admission. The univariate odds ratio of maternal ICU admission associated with pH1N1 illness increased to 155.28 (95% CI: 108.69-221.83). The same variables were retained in the multivariable stepwise model. Following adjustment for covariates, the multivariable OR was 131.25 (95% CI: 88.21-195.28). This risk estimate, however, is likely to be unreliable, as case-patients were more likely to have complete information regarding ICU admission than non-case patients.

Of the 8 women with pH1N1 illness who died, 6 gave birth to a live infant, and 4 of these 6 infants survived (Table 6). Two of the eight women died prior to a live birth, and therefore had no matching record in the birth registry. The age of deceased women ranged from 22 to 31 years, and the gestational age of their fetus at the time of their influenza infection ranged from 11 to 35 weeks. Seven of the eight deceased women were infected with pH1N1 within the first 4 months of the pandemic. Data on co-morbidities is incomplete, but at least 2 of the 8 deceased women were obese, and 2 had asthma. Of the 8 deceased women, 6 reportedly received treatment with oseltamivir during their illness, however, only 2 of these women appear to have received it within 48 hours of symptom onset. None of the 8 women had received influenza vaccine specific for pH1N1; 7 of the 8 had illness onset prior to the availability of vaccine.

**Table 6:** Characteristics of maternal deaths among women with pandemic influenza illness during pregnancy.

Age	Race	Onset of symptoms	Hospital admission date	Gestational age at time of illness	Interval between onset and death (days)	BMI	Comorbidities	Oseltamivir Treatment [Y/N, (days after onset)]	DOB of Infant	Infant outcome
30	W-H	5/28/09	6/4/09	11	25	dk	obesity asthma	N	n/a	Fetal loss
25	B	6/11/09	6/16/09	27	15	42.9	obesity, diabetes	Y [9]	6/17/09	Survived
22	W	6/23/09		26	10	dk		dk	7/02/09	D-7/2/09
25	W	7/17/09		35	11	dk	asthma	Y [5]	7/19/09	Survived
22	W-H	7/26/09	7/29/09	27	25	dk		Y [12]	8/20/09	Survived
31	A	8/4/09	8/8/09	13	9	dk		Y [9]	n/a	Fetal loss
24	W	8/5/09	8/6/09	32	54	23.7		Y [2]	8/07/09	Survived
22	W-H	11/8/09	11/18/09	33	30	dk	none	Y [1]	11/18/09	D-11/22/09

B=Black, W=White, A=Asian, H=Hispanic, dk=don’t know, n/a=not applicable, D=Deceased, DOB=date of birth

To address the possibility that some pregnant women with pH1N1 illness during the study interval were not reported to FLDOH, we searched fields in the birth record for text strings containing “H1N1”. We identified 41 birth records with “H1N1” text appearing in the record. Of these 41 births, 9 were from confirmed cases of pH1N1 ascertained through surveillance, and 32 were from women not contained in the notifiable disease database. In 15 of the 32 records, the “H1N1” text appears to relate to provision of H1N1 vaccine or prophylaxis, leaving 17 remaining records which may represent pH1N1 infections among pregnant women which were not otherwise identified through routine surveillance and case reporting. We conducted additional analysis adding these 17 pregnant women and births to confirmed pH1N1 cases, and excluding the 15 pregnant women and births for which H1N1 may refer to vaccine or chemoprophylaxis. The results of these analyses were not substantially different from previous results, and did not alter any of our conclusions; therefore, we did not utilize this approach when presenting the primary results of our analysis. Similarly, results did not differ notably when excluding or including birth records from adopted children; therefore, results are presented for analyses following exclusion of these records. 

Based on the available sample size and assuming an alpha-level of 0.05, the study analysis had 80% power to detect ORs of approximately 1.50 or greater for most of the risk factors presented in Table 1. For less common risk factors and for the primary adverse birth outcomes presented in Table 3 with approximately 10% frequency in the birth cohort (e.g. low birth weight and premature birth) the analysis had 80% power to detect ORs of approximately 1.75 or greater.

## Discussion

We observed a consistent, and statistically significant association between pH1N1 illness during pregnancy and adverse neonatal and maternal outcomes, including low birth weight, preterm birth, neonatal ICU admission, infant death, and maternal ICU admission. The observed associations with pH1N1 illness remained significant after adjusting for other observed and known risk factors for adverse neonatal and maternal outcomes. As the disease surveillance data sources used for this analysis are more likely to detect case-patients with more severe influenza illness, our findings apply more toward outcomes associated with more severe influenza illness, rather than influenza infection in general.

While several previous case series have described neonatal outcomes of pH1N1 infected women [5,8,11,12,27–30] few past reports used robust comparison groups or cohort designs to permit estimation of risk for adverse neonatal outcomes. Our estimates of risk for adverse neonatal outcomes are generally consistent with those previously reported by Pierce, et al, using a similar cohort design from the United Kingdom [31]. Methodological differences likely account for the slight differences in the magnitude of risk estimates between the 2 studies. However, in both studies, illness with pH1N1 during pregnancy was consistently and significantly associated with low and very low birth weight, late and early pre-term birth, and infant death, after adjusting for other factors.

In addition to the adverse neonatal outcomes we observed, the pandemic had a striking impact on maternal mortality in Florida, as elsewhere. Data from the Florida Pregnancy-Associated Mortality Review [32] indicate 58 pregnancy related deaths during 2009 [33]. The pregnancy-related mortality ratio (PRMR) in Florida for 2009 was 26.2 deaths per 100,000 live births, representing the largest annual PRMR in the previous ten years. This contrasts to the most recent national PRMR estimate available of 15.1 for the period 2006-2007 [34]. Of the 58 pregnancy related deaths in Florida in 2009, 15 (25.9%) were related to infection. This contrasts to the previous 10 year period in which infection accounted for approximately 10% of pregnancy related deaths. Of the 15 infection related deaths, 13 of these were associated with acute respiratory illness, and 8 were confirmed pH1N1 cases identified in our notifiable disease surveillance database, yielding a pH1N1 cause-specific PRMR of 3.6 in Florida during 2009. This compares to a pH1N1 cause-specific maternal mortality ratio of 4.3 reported from California [10] and 3.0 reported from Turkey [7]. Of the 8 pH1N1 case-patients in our analysis that died, only 2 received treatment with neuraminidase inhibitors within 48 hours of illness onset. Delayed antiviral treatment has repeatedly been identified as a risk factor for maternal ICU admission and death [5,8,10,11]. 

We identified younger age, racial and ethnic minorities, indicators of lower socio-economic status (SES), and obesity as risk factors for pH1N1 illness severe enough to be detected through our surveillance. Others have noted both an individual and ecological-level association between hospitalization for pH1N1 and indicators of low SES [35]. One possible explanation for the association with lower SES observed in our study is that surveillance case reporting was somehow biased toward reporting illness among those on public assistance. We have no evidence that surveillance case reporting was biased in this way. We also found that urban residence and previous live births were risk factors for illness. Increased risk associated with urban residence and presence of young children in the household are consistent with what is known of influenza transmission and what was observed in the United States during this pandemic [15]. 

Obesity was both an observed risk factor for recognized and reported pH1N1 illness, as well as a risk factor for poor maternal outcomes among pregnant women, as indicated by ICU admission. Obesity may have important effects on immune response which leave obese individuals more susceptible to infection [36–38]. The observed association between obesity and pH1N1 illness in this cohort, however, should be interpreted with caution. Due to the nature of surveillance and case reporting used to ascertain cases, our data likely underestimate the total number of pregnant women with pH1N1 infection and overestimate the proportion of pregnant women with severe illness. Thus the observed association with obesity likely relates at least partly to risk of more severe illness detected by our surveillance, rather than risk of infection. Coleman, et al previously concluded that obesity was not an independent risk factor for medically attended influenza, among adults with acute respiratory illness in a population based study [39]. 

Others have previously identified obesity as a risk factor for severe illness with pH1N1 [16–18,40,41]. Both Louie and Morgan reported that hospitalization and death from pH1N1 were associated with morbid obesity, however, both studies excluded pregnant women. Kwong et al, noted hospitalizations for respiratory illness during influenza season were consistently associated with obesity over several years, however this analysis also excluded pregnant women. Morbid obesity is associated with reduced fertility[42] making the condition less common among pregnant women than the general, reproductive-aged female population. There were a very small number of women with pH1N1 illness in our cohort with BMI ≥ 40, limiting our analysis of the effect of morbid obesity on maternal and neonatal outcomes. Obesity is also associated with certain complications and adverse outcomes of pregnancy such as gestational diabetes, preeclampsia, macrosomia, still birth, infant death, cesarean section delivery, and possibly certain birth anomalies and elective pre-term delivery [20,21,43,44]. Therefore, an important strength of our study was the availability of data on pre-pregnancy BMI for most cohort members, which allowed us to control for BMI when estimating risk for adverse neonatal outcomes associated with pH1N1 illness. Nevertheless, we observed no interaction between pH1N1 illness and pre-pregnancy obesity, as it relates to risk of maternal ICU admission, early preterm birth, or infant mortality. 

Pierce et al, used a similar approach to ours, however, the comparison cohort they used was smaller and taken exclusively from the pre-pandemic period [31]. The disadvantage of using a comparison group from the pre-pandemic period is that, while there is no chance any of those in the comparison cohort were infected with the pandemic virus, it fails to account for women who may have been infected with seasonal influenza during pregnancy, and the impact this may have had on maternal and fetal outcomes. As imperfect as disease surveillance was in the initial months of the pandemic, it was better at detecting individual cases of influenza in Florida than during the pre-pandemic period, when individual cases of seasonal influenza were not reportable to the health department. For this reason, we chose the primary comparison group for our analysis to coincide with the period of infection and births among the pH1N1 case-patients in our analysis. Nevertheless, results of secondary analysis using a cohort from the pre-pandemic period did not substantially alter our findings. 

By the end of 2009, an estimated 25% of residents in some areas of Florida were believed to have been infected with pH1N1, with the highest rates of infection noted among school-aged children 5-17 years, and infection rates approaching 50% among young adults 18-24 years [45]. Seroprevalence studies conducted elsewhere suggest similar findings [46–48], however, work from Australia suggests that infection rates may have been lower in pregnant women [46]. If the Florida estimate of 25% infection by the end of 2009 also holds true for pregnant women, then far more than the 194 women we observed would have been infected with pH1N1 during pregnancy. We attempted to overcome limitations in the case ascertainment by also searching text fields for H1N1 in the birth record, and this did not substantially alter our results. Nonetheless, the results we present are likely to represent those associated with the severe end of the spectrum of influenza illness, as women with mild illness during pregnancy may have gone undiagnosed and unrecognized in our analysis. 

The study is limited by imperfect disease surveillance and case ascertainment of all women with pH1N1 illness during pregnancy. Therefore, our findings are only generalizable to the more severe pH1N1 illness detected through our surveillance, not to pH1N1 infection in general. Imperfect disease surveillance may have led to both random and differential misclassification bias of pH1N1 illness, which was both an outcome and exposure variable in this analysis, possibly resulting in some bias in the risk estimates. This study was also limited by lack of comparative data for women without a live birth and missing data on pre-pregnancy BMI for approximately 6% of the women. Data available in the birth record regarding maternal morbidity, (e.g. ICU admission), also appears to be incomplete, somewhat limiting our conclusions regarding maternal outcomes. Prospective studies may help address all of these limitations. However, the advantages of our study over past reports include a very large comparison group with extensive information available from the birth record to facilitate control for confounding factors, as well as longer follow-up to allow ascertainment of child mortality, through the birth record, several months after birth. 

## Conclusions

The present study uses a population-based cohort design to add to and further characterize the previously described risk of adverse maternal and neonatal outcomes associated with pH1N1 illness. In conclusion, we found increased risk of severe adverse maternal and neonatal outcomes associated with pH1N1 illness during pregnancy. Pregnant women should continue to be regarded as a high risk group for adverse outcomes of influenza virus infection and should continue to be targeted for enhanced prevention activities through vaccination and appropriate chemoprophylaxis and treatment for influenza, consistent with current recommendations [3,49,50].
